# Wandering spleen presenting in the form of right sided pelvic mass and pain in a patient with AD-PCKD: a case report and review of the literature

**DOI:** 10.1186/s13256-024-04580-6

**Published:** 2024-05-25

**Authors:** Yitagesu aberra shibiru, Sahlu wondimu, Wassie almaw

**Affiliations:** Addis Ababa University, Addis Ababa, Ethiopia

**Keywords:** Wandering spleen, Splenic torsion, Splenic infarction, AD-PCKD, Splenectomy, Splenopexy

## Abstract

**Background:**

Wandering spleen is a rare clinical entity in which the spleen is hypermobile and migrate from its normal left hypochondriac position to any other abdominal or pelvic position as a result of absent or abnormal laxity of the suspensory ligaments (Puranik in Gastroenterol Rep 5:241, 2015, Evangelos in Am J Case Rep. 21, 2020) which in turn is due to either congenital laxity or precipitated by trauma, pregnancy, or connective tissue disorder (Puranik in Gastroenterol Rep 5:241, 2015, Jawad in Cureus 15, 2023). It may be asymptomatic and accidentally discovered for imaging done for other reasons or cause symptoms as a result of torsion of its pedicle and infarction or compression on adjacent viscera on its new position. It needs to be surgically treated upon discovery either by splenopexy or splectomy based on whether the spleen is mobile or not.

**Case presentation:**

We present a case of 39 years old female Ethiopian patient who presented to us complaining constant lower abdominal pain especially on the right side associated with swelling of one year which got worse over the preceding few months of her presentation to our facility. She is primiparous with delivery by C/section and a known case of HIV infection on HAART. Physical examination revealed a right lower quadrant well defined, fairly mobile and slightly tender swelling. Hematologic investigations are unremarkable. Imaging with abdominopelvic U/S and CT-scan showed a predominantly cystic, hypo attenuating right sided pelvic mass with narrow elongated attachment to pancreatic tail and absent spleen in its normal position. CT also showed multiple different sized purely cystic lesions all over both kidneys and the pancreas compatible with AD polycystic kidney and pancreatic disease.

With a diagnosis of wandering possibly infarcted spleen, she underwent laparotomy, the finding being a fully infarcted spleen located on the right half of the upper pelvis with twisted pedicle and dense adhesions to the adjacent distal ileum and colon. Release of adhesions and splenectomy was done. Her post-operative course was uneventful.

**Conclusion:**

Wandering spleen is a rare clinical condition that needs to be included in the list of differential diagnosis in patients presenting with lower abdominal and pelvic masses. As we have learnt from our case, a high index of suspicion is required to detect it early and intervene by doing splenopexy and thereby avoiding splenectomy and its related complications.

## Introduction

Wandering spleen is a rare clinical entity characterized by hypermobility of the spleen as a result of absence or abnormal laxity of its suspensory ligaments which in turn can be congenital or precipitated by a number of risk factors like repeated pregnancy, trauma, surgery or connective tissue disorder. The spleen therefore migrates from its normal left hypochondriac position, to other parts of the peritoneal cavity especially the pelvis [3]. Since the first case report in 1667, there have been less than 600 cases reported in the literature so far [1, 3].

Wandering spleen can have different clinical presentations ranging from asymptomatic incidental finding on imaging to features of acute abdomen as a result of complete torsion of the pedicle and total infarction of the spleen or complete obstruction of adjacent hollow viscus due to pressure effect. Less dramatic presentation includes chronic lower abdominal pain, swelling and symptoms of partial obstruction of bowel especially of the colon [3–6].

Diagnosis is confirmed by imaging usually abdominal ultrasound or CT which reveals that the spleen is absent from its normal anatomical position but seen somewhere else in the new location within the peritoneal cavity [3, 9, 10]. Once diagnosed, surgical intervention is required either by splenopexy or splenectomy depending on the viability of the organ [3, 5] and can be done laparoscopically or by laparotomy.

Owing to its rarity, a high index of suspicion is required and this condition should always be considered as a possible differential diagnosis in patients presenting with lower abdominal swelling and pain. We present this case to share our experience in diagnosing and managing such a rare pathology and once again bring it to the attention of fellow clinicians handling this sort of abdominal conditions.

## Case summary

Our patient is a 39 years old female Primi-para Ethiopian, who presented with lower abdominal dull aching pain of one-year duration which got worse over the last few months associated with right lower abdominal swelling, easy fatigability, LGIF, loss of appetite and weight. She is a known case of RVI on HAART for the past 18yrs and hypertensive for the last 8 years for which she was taking enalapril and atenolol. Her only child was delivered by C/section 10 years ago.

**Fig. 1:**
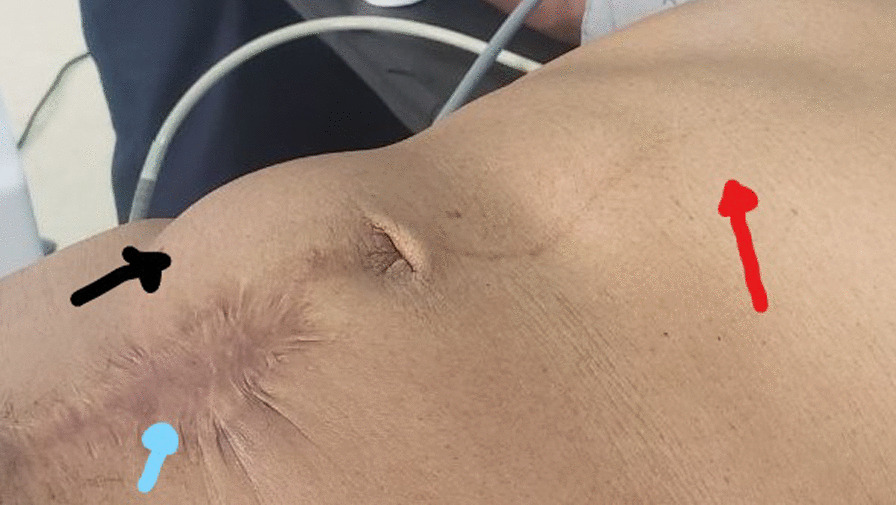
Black arrow shows the splenic mass, red arrow shows the stomach, cyan arrow shows previous CS scar

On examination**,** she looked chronically sick with her vitals in the normal range. The abdomen was flat with a lower midline surgical scar and a visible round mass on the right paraumblical and lower quadrant areas. The mass was well defined, smooth surfaced, slightly tender and mobile (Fig. 1—black arrow).

### Laboratory

Her hematologic tests revealed WBC of 8.7 × 103, Hgb of 12.3 and PLT count of 544 × 10^3^. Serum electrolyte and liver function tests were all in the normal range. Creatinine was 1.4 mg/dl.

### Imaging

#### Abdominal ultrasound

Multiple bilateral renal, liver and pancreatic cysts. An ehcocomplex mainly hypoechoic, 13 cmx8cm well defined right sided abdomino-pelvic mass, with absent color Doppler flow. Spleen was not visualized in its normal anatomic site.

#### Contrast enhanced abdomino-pelvic CT

**Fig. 2:**
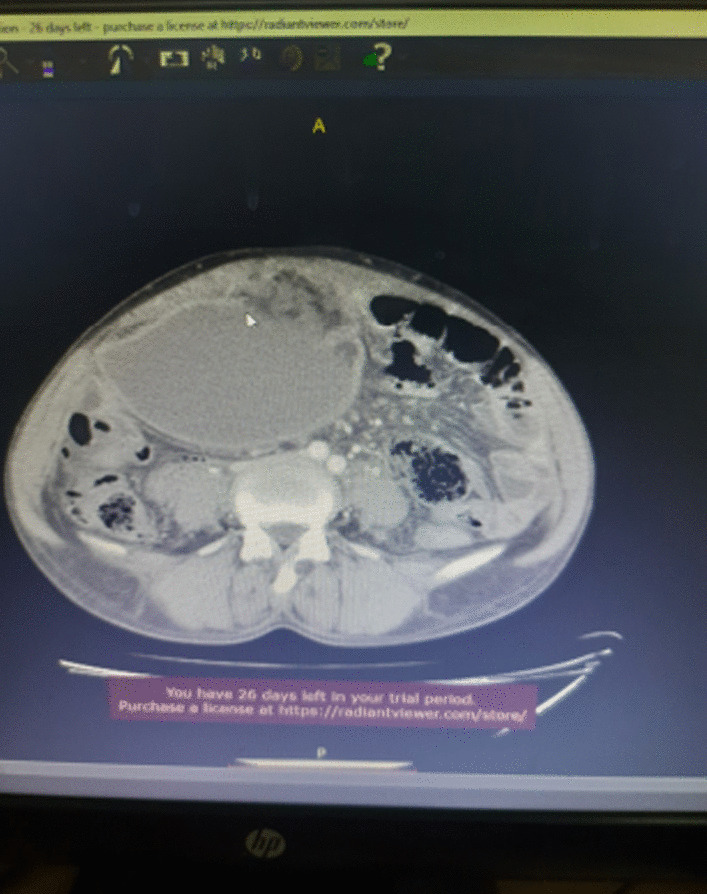
Infarcted spleen

**Fig. 3:**
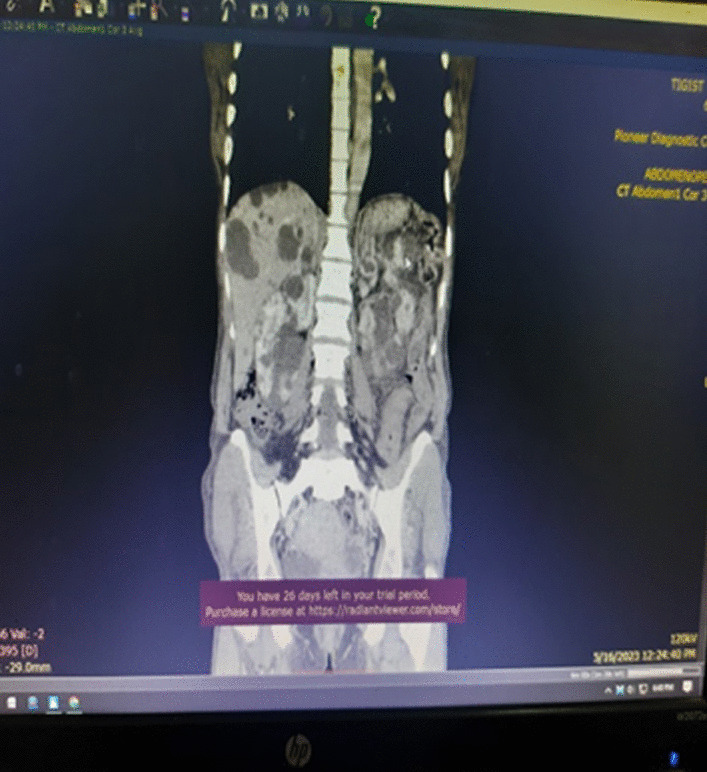
Absent spleen in the splenic fossa

**Fig. 4:**
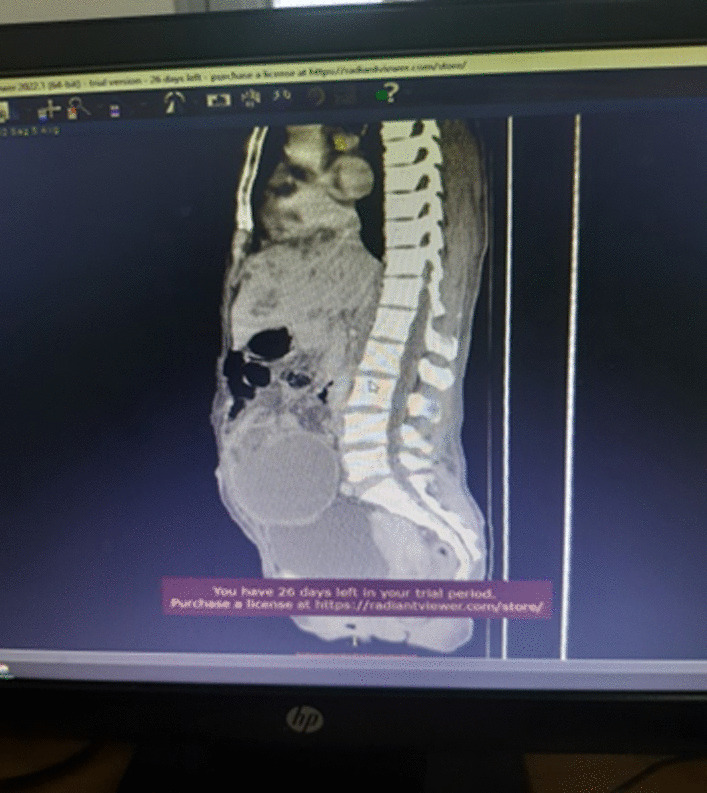
Spleen seen in the abdomino-pelvic cavity

Described the mass as a hypoattenuating, well circumscribed lesion with no contrast enhancement located at right abdomino pelvic cavity (Fig. 2). Its long torsed pedicle could be traced to the region of the tail of the pancreas and the spleen was missing from its normal location. (Fig. 3) Majority of the renal parenchyma is almost replaced with different sized cystic lesions with imperceptible wall causing bilateral renal enlargement. (Fig. 3) The liver and the pancreas too is filled with similar cysts. The portal vein were not visualized and replaced by periportal enlarged collateral vessels. (Figs. 3, 4).

### Management

**Fig. 5:**
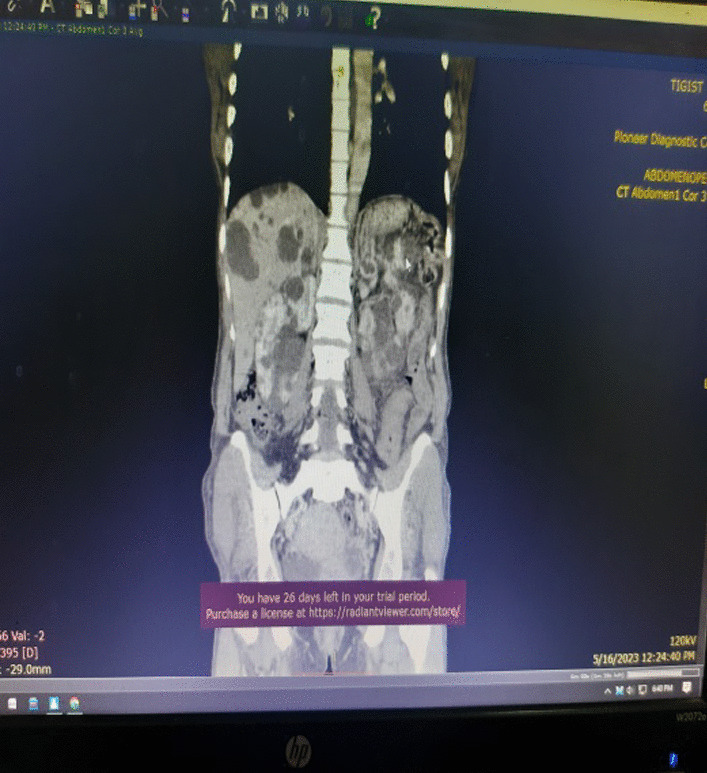
The intra-op picture of our patient upon exploration

With a diagnosis of wandering spleen located in the right abdomino pelvic region with torsion of the pedicle and infarction, she was admitted and underwent laparotomy. Intraoperatively, dense adhesion encountered between the anterior abdominal wall, omentum, the wandering spleen and small bowel. The spleen was whitish, distended and grossly infarcted with its long stalk torsed > 360°. (Fig. 5) Adhesions were gently released and splenectomy done. The splenic mass was sent for biopsy.

She was discharged on the 3rd postoperative day and her post-operative course was uneventful. She was seen after a month on follow up clinic with no report of complication. Her biopsy result showed splenic tissue. She got her pentavelant vaccine on the third week.

## Discussion

**Fig. 6:**
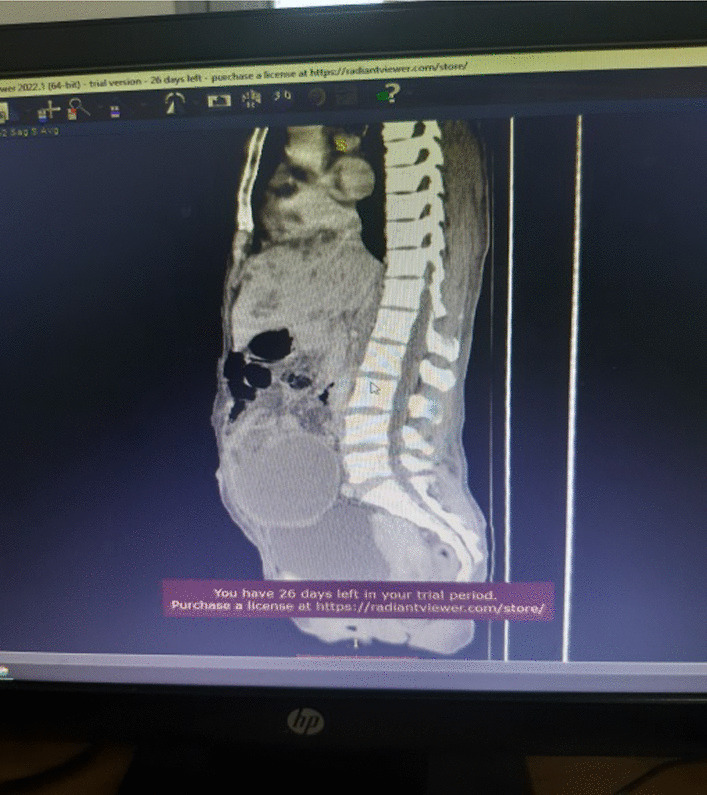
Absent spleen in the splenic fossa

**Fig. 7:**
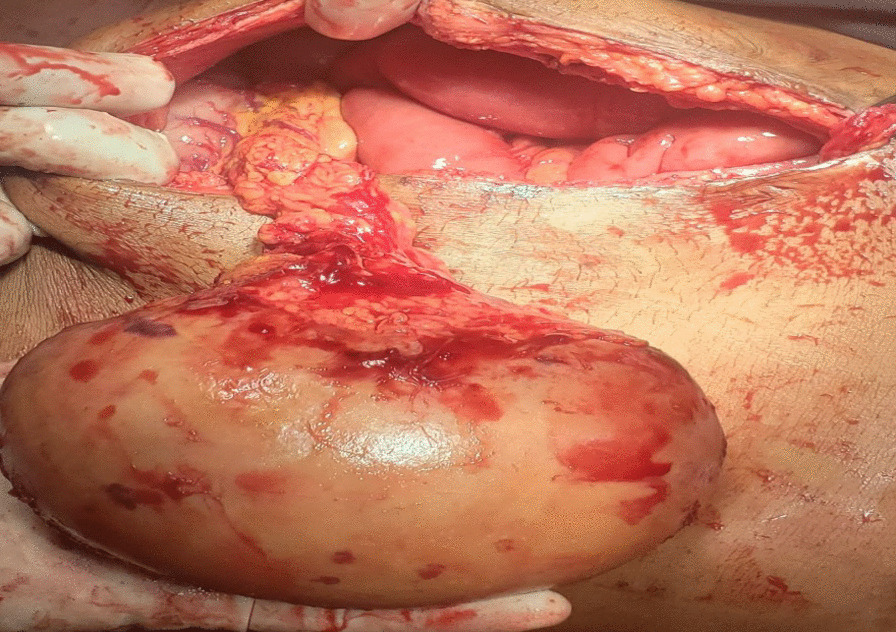
Spleen seen in the abdomino-pelvic cavity

- Wandering spleen is a rare clinical entity characterized by splenic hypermobility from its left hypochondriac position to any other abdominal or pelvic position caused by absent or abnormal laxity of the suspensory ligaments [1, 2].
- The first case of wandering spleen was reported by Von Horne in 1667. So far less than 600 cases are reported world wide [1, 3].
- Anatomically a normal spleen is found in the left hypochondriac region suspended by ligaments to the stomach, kidney, pancreas, colon and left hemi-diaphram by the gastrosplenic, splenorenal, pancreaticosplenic, splenocolic, splenophreni ligaments and presplenic folds [1]. Our patient presented with RLQ palpable abdominal mass which is against the commonest presentation being in the LLQ of the abdomen (Fig. 1).
- It could result from either a developmental failure of the embryonic septum transversum to fuse properly with the posterior abdominal wall which results in absent/lax ligaments [4] or from acquired conditions that result in lax suspensory ligaments as in pregnancy or connective tissue disorders [3]. The spleen is found in any quadrant of the abdomen or the pelvis though mostly in the left quadrants attached only by a long and loose vascular pedicle. Our patient presented with RLQ mass.
- It is mostly seen in multiparous women [4] though the incidence is found to be nearly equal in both sexes in the prepubertal age group [3]. Our patient was a Para 1 mother and presented with 01 year history of abdominal pain which got worse in the past 06 months. Otherwise she had no any other pressure symptoms. She had visible umbilical area mass which was mobile up on examination
- Wandering spleen can have different presentation ranging from asymptomatic incidental finding on imaging or upon surgical exploration for other surgical conditions to a presentation that mimics acute abdomen [3, 5]. Mostly it presents as an on and of type acute/ subacute non-specific abdominal pain due to torsion and spontaneous de-torsion of the loose splenic pedicle [3, 4]. This chronic torsion results in congestion and splenomegaly [3, 5]. Hence patients could have palpable mobile mass [6] which is the typical presentation of this patient. The other presentations are usually related to the mass effect of the enlarged spleen and patients could present with GOO, bowel obstruction, pancreatitis and urinary symptoms [3, 6].
- In some cases it is reported to be associated with some other disorders like gastric volvulus [7] and distal pancreatic volvulus [8].
- Ultrasound is one of the imaging modalities to investigate patients whom we suspect had wandering spleen. It usually shows absent spleen in the splenic fossa and a comma shaped spleen in the abdomen or pelvis [9]. Doppler study might help us see the vascular condition and ads up to a better preoperative plan. CT scan shows absence of the spleen in the left upper quadrant, ovoid or comma-shaped abdominal mass, enlarged spleen, a whirled appearance of non-enhancing splenic vessels and signs of splenic hypo-perfusion: homogenous or heterogeneous decreased enhancement depending on the degree of infarction [3, 9, 10].
- Our patient was scanned with US and showed 13*8 cm large midline abdomino-pelvic well defined oval mass which was predominantly solid with areas of cystic component with absent color Doppler flow. Otherwise the spleen was not visualized in the splenic fossa. Bilateral kidney and liver has multiple different sized cystic lesions. With this image Abdomino-pelvic CT was done and shows spleen is located in the lower abdomen and appears to have torsed vascular pedicle and the whole splenic parenchyma is hypodense and no enhancement seen. Majority of the renal parenchyma is almost replaced with different sized cystic lesions with imperceptible wall causing bilateral renal enlargement. The whole liver is filled with cystic lesions with imperceptible wall. The portal veins were not visualized and replaced by periportal enlarged collateral vessels (Figs. 6, 7).
- Usually surgical management is the rule once a patient is diagnosed with wandering spleen [3, 5]. Most patients; 65% as reported in some studies will have torsion of the vascular pedicle at some point of their life [5, 6]. Hence splenopexy or splenectomy shall be considered when a wandering spleen is found incidentally up on surgical exploration for some other purposes [6]. Complicated wandering spleen like infarcted, signs of hypersplenism, huge in size and splenic vein thrombosis needs splenectomy while others can be managed with splenopexy [3, 5, 6]. Nowadays though laparoscopic technique is the gold standard, open technique can be used for splenopexy and splenectomy [3, 5].
- Partial infraction of a wandering spleen might necessitate partial splenectomy and splenopexy or splenectomy and splenic implantation [6, 11].
- The spleen might get fixed by different methods [8, 9].
  1. Simple splenic fixation involves simple tacking the splenic capsule to the peritoneum
  2. Retroperitoneal pouch splenopexy- Tissue [11, 12]/Mesh splenopexy (sandwich technique) [13].
  3. Omental and peritoneal pouch splenic fixation [14].
- In our case, Spleen was absent from the normal anatomic splenic fossa and the spleen in the abdomino-pelvic area looks infarcted. Hence she was managed with splenectomy and the patient was extubated on table and having a stable postoperative course*.*

## Conclusion

Wandering spleen is a rare form of splenic pathology. Such a rare pathology presents commonly as an acute torsion with infarction. Spleen in the RLQ with chronic torsion and infarction is a very rare presentation for wandering spleen. In addition there is no report of such a presentation in a patient with AD-PCKD.

## Recommendation

We recommend Clinicians to consider wandering spleen in their differential diagnosis in a patient presenting with RLQ abdominal mass and chronic abdominal pain.

## Data Availability

Data related with this case report is available at Addis ababa university, Tikur Ambesa Tertiary Hospital.

## Abbreviations

AD-PCKD: Autosomal dominant polycystic kidney disease
BP: Blood pressure
LGIF: Low grade intermittent fever
HAART: High active anti-retroviral therapy
RLQ: Right lower quadrant
RVI: Retro viral infection
HTN: Hypertension
WBC: White blood cell count
PLT: Platelet
HGB: Hemoglobin
